# Calcium Binding-Mediated Sustained Release of Minocycline from Hydrophilic Multilayer Coatings Targeting Infection and Inflammation

**DOI:** 10.1371/journal.pone.0084360

**Published:** 2014-01-07

**Authors:** Zhiling Zhang, Camilla A. Nix, Utku K. Ercan, Jonathan A. Gerstenhaber, Suresh G. Joshi, Yinghui Zhong

**Affiliations:** 1 School of Biomedical Engineering, Science and Health Systems, Drexel University, Philadelphia, Pennsylvania, United States of America; 2 Surgical Infection Program, Department of Surgery and Department of Microbiology and Immunology, Drexel University College of Medicine, Philadelphia, Pennsylvania, United States of America; University Hospital of the Albert-Ludwigs-University Freiburg, Germany

## Abstract

Infection and inflammation are common complications that seriously affect the functionality and longevity of implanted medical implants. Systemic administration of antibiotics and anti-inflammatory drugs often cannot achieve sufficient local concentration to be effective, and elicits serious side effects. Local delivery of therapeutics from drug-eluting coatings presents a promising solution. However, hydrophobic and thick coatings are commonly used to ensure sufficient drug loading and sustained release, which may limit tissue integration and tissue device communications. A calcium-mediated drug delivery mechanism was developed and characterized in this study. This novel mechanism allows controlled, sustained release of minocycline, an effective antibiotic and anti-inflammatory drug, from nanoscale thin hydrophilic polyelectrolyte multilayers for over 35 days at physiologically relevant concentrations. pH-responsive minocycline release was observed as the chelation between minocycline and Ca^2+^ is less stable at acidic pH, enabling ‘smart’ drug delivery in response to infection and/or inflammation-induced tissue acidosis. The release kinetics of minocycline can be controlled by varying initial loading, Ca^2+^ concentration, and Ca^2+^ incorporation into different layers, enabling facile development of implant coatings with versatile release kinetics. This drug delivery platform can potentially be used for releasing any drug that has high Ca^2+^ binding affinity, enabling its use in a variety of biomedical applications.

## Introduction

Infection and inflammation are common coexisting issues that seriously affect the functionality and longevity of a variety of medical implants including biosensors [1], [2], neural prostheses [3], [4], catheters [5], [6], and stents [7], [8], etc. Pathogenic bacteria often colonize and adhere to the implant surface to form a biofilm that causes persistent and chronic infections necessitating implant removal [9]–[12]. Conventional systemic antibiotic therapy is ineffective in killing bacteria within a biofilm [10]–[12]. Bioactive coatings capable of releasing antibacterial agents can deliver high doses of antibiotics during the “decisive period” (6 h post implantation) to inhibit initial bacterial adhesion, which is crucial for biofilm formation [10], [11]. Subsequent continued release over weeks to months is desirable to ensure that tissue integration can occur before bacterial adhesion [10]. Implant-associated inflammation is caused by tissue injury and the foreign body reaction [13], [14], and results in fibrotic encapsulation that compromises implant performance. For implants that are intended to monitor, record, or stimulate within the body, such as glucose sensors and neural electrodes, the fibrous capsule limits device-tissue communication. For implants that are intended to repair or replace lost tissue functions, such as stents and catheters, inflammation induces stent restenosis and obstruction of catheters. Systemic administration of anti-inflammatory agents is often ineffective in treating local fibrotic encapsulation and long-term treatment can lead to serious side effects such as gastrointestinal symptoms (ulcers, bleeding, and perforation) [15], heart failure [16], and renal failure [17]. Thus, sustained local drug delivery from medical implants is desirable to effectively treat inflammation while limiting deleterious side effects. However, methods for co-delivery of both antibiotics and anti-inflammatory agents at physiologically relevant concentrations and time scale are limited because the drugs have different physiochemical properties [18].

Minocycline Hydrochloride (MH), a tetracycline derivative, is a broad-spectrum antibiotic and effective anti-inflammatory drug that is widely used clinically to treat infection and inflammation [19], [20], making it a promising drug candidate to combat implant-associated infection and inflammation by local delivery. In designing suitable coatings for medical implants, the thickness and biocompatibility of the coating are important design considerations. Thin coatings are preferable to avoid additional tissue injury and ensure effective device-tissue communication. Moreover, hydrophilic coatings have been shown to minimize nonspecific protein adsorption and inflammatory/immune cell adhesion that would exacerbate the foreign body response [8], [13], [21], [22]. However, small molecules that are highly soluble in water, including MH (MW 493.94 Da), diffuse very quickly from hydrophilic thin films (on a scale of minutes to hours) [23], [24], due to the lack of strong interactions between the small molecule and polymer layers.

Layer-by-layer (LbL) self-assembly technique is a versatile method to construct thin film coatings via alternative adsorption of building blocks driven by weak molecular interactions such as electrostatic force and hydrogen bonding [25]. The resulting films typically have a thickness ranging from nanoscale to microscale. However, the release of hydrophilic drugs from LbL coatings is normally very fast. Rapid penetration of water into the thin coatings combined with a lack of strong forces holding the biomolecules in place usually result in complete release of loaded drug within a few days. So far only one coating design for concurrent sustained delivery of both antibiotic and anti-inflammatory drugs was reported [18], from which the release of hydrophilic antibiotic vancomycin lasted from 4 hr to 2.3 days, and the release of hydrophobic anti-inflammatory drug diclofenac lasted from 1.7 to 14 days. In this study, we report the integration of a novel calcium binding-mediated drug delivery mechanism with electrostatic layer-by-layer (LbL) assembly to create biocompatible, hydrophilic, and nanoscale thin coatings capable of sustained release of physiologically relevant levels of MH for over 35 days. Moreover, we were able to achieve a high drug density of 645 µg/mm^3^ from 8 trilayers of LbL film. In contrast, the highest drug density reported so far is 220 µg/mm^3^ from 60 tetralayers of LbL film [18], [26], [27]. We postulate that the high drug loading capacity and sustained release reported here is due to the strong Ca^2+^ binding-mediated interactions. MH as a tetracycline derivative can chelate Ca^2+^ ions, without affecting its biological activity [28]. Dextran sulfate (DS) is a biodegradable polysaccharide that also has a high binding affinity for Ca^2+^ due to its numerous negatively charged sulfate groups [29], [30]. Utilizing this property, we used Ca^2+^ as the linker to attach MH to DS. We further found that DS can form electrostatic layer by layer (LbL) assembly with gelatin type A (GA), a positively charged biodegradable natural polymer derived from collagen. Multilayers of DS-Ca^2+^-MH conjugate/GA were successfully constructed to form hydrophilic nanoscale thin coatings. We hypothesize that MH loading and release in the LbL assembly is mediated by calcium binding. Sustained MH release over 35 days was obtained from only 8 trilayers of LbL films with a thickness of 402 nm. We found that Ca^2+^-mediated MH release was pH-responsive, probably because the binding affinity of tetracycline for Ca^2+^ decreases with pH [31], [32]. Reduced extracellular pH (tissue acidosis) is commonly found under pathophysiological conditions such as tissue injury, inflammation, or infection [33]–[36]. Therefore, pH-responsive release of MH will potentially enable ‘smart’, tissue response-regulated drug delivery at the implant-tissue interface. MH release from these films significantly inhibited biofilm formation by bacteria from seven different strains including multi-drug resistant *Acinetobacter baumannii* (*A. baumannii*), and inhibited the activation of inflammatory macrophages. The combination of hydrophilic coatings that minimize bacterial and inflammatory cell adhesion with sustained release of anti-bacterial and anti-inflammatory MH holds great potential to combat implant-associated infection and inflammation.

## Materials and Methods

### Materials

All the chemicals were obtained from Sigma-Aldrich and used without further purification. *Escherichia coli* (ATCC 25922), Escherichia coli O157∶H7, *Acinetobacter baumannii* (ATCC 19606), *Staphylococcus aureus* (ATCC 25923), methicillin-resistant *Staphylococcus aureus* USA300 (BAA 1860) and *Staphylococcus epidermidis* (ATCC 12228) strains were purchased from American Type Culture Collection (ATCC, Manassas, VA). A multi-drug resistance *Acinetobacter baumannii* clinical isolate (referred as *A. baumannii* #22) was locally isolated from a hospitalized patient having invasive infection following a protocol approved by the Institutional Review Board of Drexel University. The study involves retrospective collection of isolates from clinical laboratory of the hospital, and the samples were coded so that the patient's details were not revealed and identity was not determined. Investigator is granted waiver for this protocol and no consent is required.

### Preparation of FITC-GA conjugate

Fluorescein isothiocyanate (FITC)-labeled GA was prepared as reported [37]. Briefly, 100 mg GA was dissolved in 0.1 M sodium bicarbonate buffer at the concentration of 5 mg/ml, 350 µl FITC solution (10 mg/ml in DMSO) was added dropwise while stirring. Size exclusion chromatography was used to remove unreacted FITC.

### LbL film assembly and characterization

LbL self-assembled films were deposited on UV-transparent 96 well plates and black 96 well plates for characterization of MH incorporation and film growth by UV absorption and fluorescence measurement, or on silicon substrates (University Wafer) for thickness measurement. 1 mg/ml solutions of dextran sulfate (DS, MW 500,000), MH, and GA were prepared in either CaCl_2_ solution or deionized (DI) water. The concentration of CaCl_2_ solution was 7.2 mM unless specified otherwise. The substrates were first coated with polyethyleneimine (PEI) as an initiating positively charged base layer, followed by alternating immersion in solutions of DS, MH, and GA with or without Ca^2+^ for 10 min. The excess molecules were removed by rinsing the substrates with DI water for 1 min between each step.

LbL film growth was monitored by UV-vis spectroscopy at 245 nm to detect MH incorporation (GA and DS have negligible absorbance at this wavelength), and fluorescence spectroscopy at an excitation wavelength of 485 nm and an emission wavelength of 535 nm to characterize FITC-labeled GA incorporation, using a Tecan M200 microplate reader (San Jose, CA). Thickness measurements were performed on films deposited on silicon wafers using a Zygo Newview 6000 optical profilometer (Middlefield, CT).

### In vitro MH release assay

LbL films were incubated in Hank's Balanced Salt Solution (HBSS) at 37°C for quantification of MH release until no detectable MH was released. Every 24 h, HBSS was removed and replaced with fresh HBSS. The amount of MH released at each time point was determined by UV absorbance at 245 nm.

### Biofilm assay

Bacterial suspensions were inoculated in 96-well plates at a density of 2×10^4^ cells per well. The wells in the plates were either pre-coated with 8 bilayers of (DS+Ca^2+^/GA+Ca^2+^) or 8 trilayers of (DS+Ca^2+^/MH+Ca^2+^/GA+Ca^2+^). Uncoated wells (polystyrene) were used as control. The biofilms were allowed to form by incubating at 37°C in a stationary incubator for 24 h [38]–[41]. For quantification of bacterial viability, the biofilm containing wells were washed three times with sterile PBS to remove loose planktonic cells, and 200 ul of XTT reagent was added and incubated with biofilm for 2 h in the dark at 37°C. The metabolic conversion of XTT into orange-colored product was measured photometrically at 492 nm using a microtiter plater reader (BioTek). Only surviving/live bacterial cells show evidence of respiration and metabolize XTT reagent to reduce it to an orange colored soluble product [42]. For visualization of live and dead bacteria, the cells were stained with LIVE/DEAD BacLight Bacterial Viability kit (Invitrogen), and examined by EVOS FL Color Imaging System (AMG).

### Anti-inflammatory bioactivity of released MH

RAW264.7 murine macrophages (kindly provided by Dr. Narayan Avadhani, University of Pennsylvania) were treated with lipopolysaccharide (LPS), fresh MH (1 µg/ml), and MH released during a 24 h period on day 32 (diluted to 1 µg/ml). After 48 h, the accumulated levels of nitrite in the cell culture medium, as an indication of NO, were measured with Griess reagent (Promega).

### Statistical analysis

Pairwise comparisons were conducted using a general linear ANOVA model and Tukey test (two-sided), and *P*<0.05 was considered statistically significant. Data are presented as mean ± standard deviations.

## Results and Discussion

### LbL multilayer film growth and characterization

**Figure 1:**
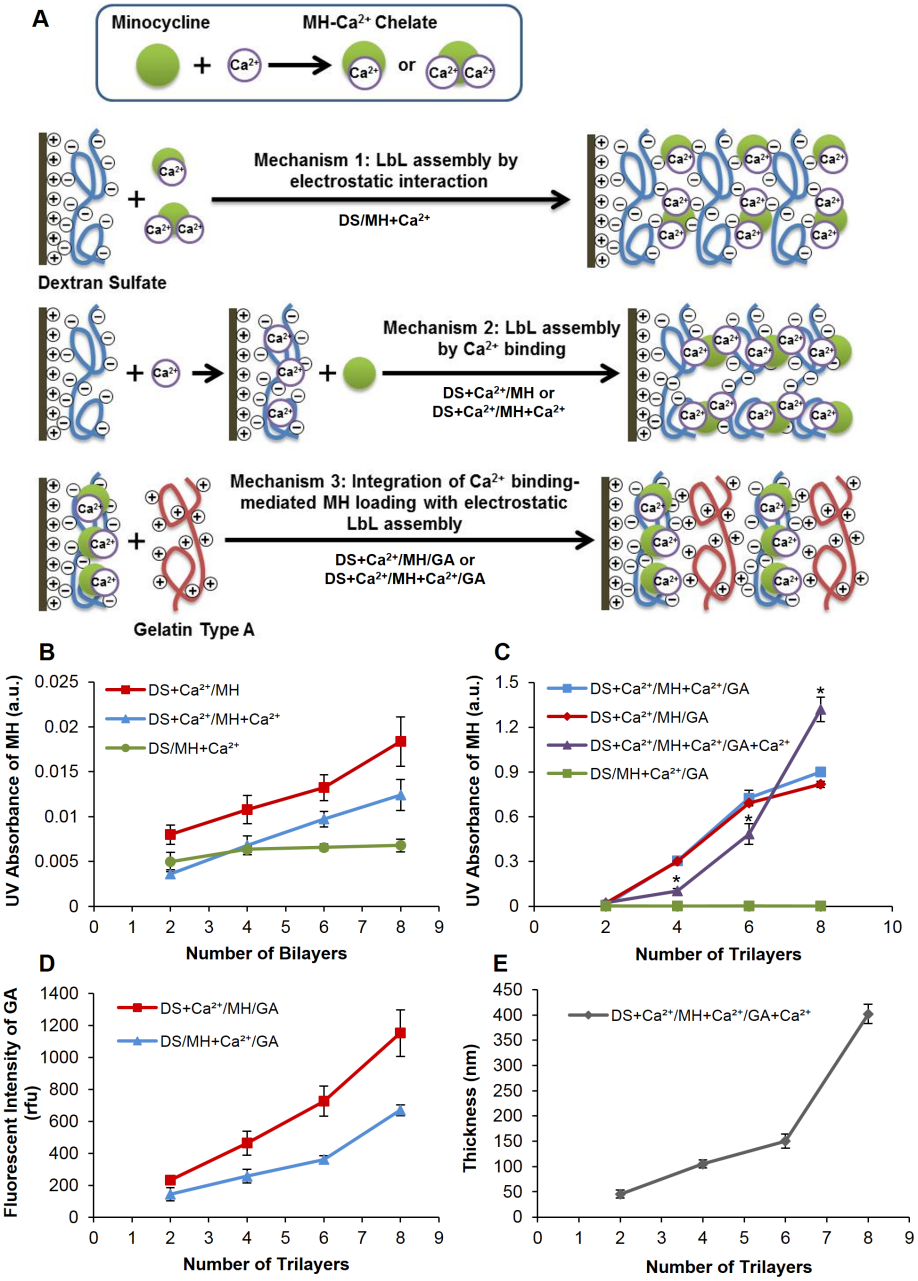
Mechanism schematic and growth of LbL assembly. (A) Schematic illustrating the mechnism of MH incorporation and LbL assembly. (B) UV absorbance of MH during DS/MH LbL assembly. (C) UV absorbance of MH during DS/MH/GA LbL assembly. *, *P*<0.05 compared with (DS+Ca^2+^/MH/GA) and (DS+Ca^2+^/MH+Ca^2+^/GA) LbL films. (D) Fluorescent intensity of FITC-GA during DS/MH/GA LbL assembly. (E) Film thickness of as a function of the number of trilayers deposited on silicon substrates. Data shown are average ±STD (n = 3).

Three different mechanisms of LbL assembly of DS and MH were explored in this study (Figure 1A). Coatings prepared by the first mechanism, by which negatively charged DS and positively charged MH-Ca^2+^ chelates were alternately adsorbed onto the wells of a UV-transparent 96 well plate, showed no increase in UV absorbance at 245 nm for MH as the number of bilayers increased (Figure 1B, green line), indicating that the chelates failed to form LbL assembly with DS. This result is not surprising since electrostatic interactions between charged small molecules and polymers are typically not strong enough for LbL assembly. In contrast, adding Ca^2+^ ions to the DS solution resulted in increased UV absorbance of MH with increased number of bilayers (Figure 1B, blue line). It is noteworthy that LbL assembly could still form even when no Ca^2+^ ions were added to the MH solution (Figure 1B, red line). This result revealed that Ca^2+^ ions in DS are required for the binding of MH to DS. One MH molecule can chelate one or two Ca^2+^ ions [28]. We speculate that MH acts as a bridge that binds to two Ca^2+^ ions in adjacent DS layers to induce LbL assembly (Figure 1A, mechanism 2). Interestingly, this result also indicates that MH could chelate DS-bound Ca^2+^, but Ca^2+^ that was already chelated by MH failed to bind to DS. We speculate that MH created steric hindrance that blocked this binding.

Drug release study of films prepared by mechanism 2 showed that only 0.34 µg/cm^2^ MH was loaded in 8 bilayers of (DS+Ca^2+^/MH) films, indicating an inefficient LbL assembly. To increase the efficiency of MH loading, we combined calcium binding-mediated MH loading mechanism with an efficient LbL assembly design (Figure 1A, Mechanism 3). GA is a positively charged biodegradable natural polymer derived from collagen. Multilayers of DS-Ca^2+^-MH conjugate/GA were successfully constructed to form hydrophilic nanoscale thin coatings. As shown in Figure 1C (blue and red lines), the use of GA to enhance LbL assembly caused a substantial increase in the loading of MH indicated by UV absorbance. GA failed to incorporate MH in the films when no Ca^2+^ was added to the DS solution (Figure 1C green line), despite successful LbL film growth indicated by increased fluorescence intensity from FITC-conjugated GA (Figure 1D, blue line). This result confirms that Ca^2+^ ions in DS layers are essential for MH loading. Taken together, these results suggest that the mechanism of MH loading was mediated by Ca^2+^ binding, rather than electrostatic interaction.

We further studied the effect of adding Ca^2+^ in GA layers on LbL film growth. The addition of Ca^2+^ to the GA layer resulted in significantly lower MH loading at 4 and 6 trilayers, but significantly higher MH loading at 8 trilayers (Figure 1C, purple line). Profilometry measurements further confirmed film growth with increased number of trilayers, with an average trilayer thickness of 50 nm for DS+Ca^2+^/MH+Ca^2+^/GA+Ca^2+^ multilayer film (Figure 1E). The thickness of (DS+Ca^2+^/MH+Ca^2+^/GA+Ca^2+^)_8_ film before and after drug release was 402±19 and 294±65 nm respectively, indicating that most coating materials still remained after MH was depleted and MH release from the LbL coating was not mediated by degradation of the LbL films.

### Effect of Ca^2+^ concentration on MH loading and release

**Figure 2:**
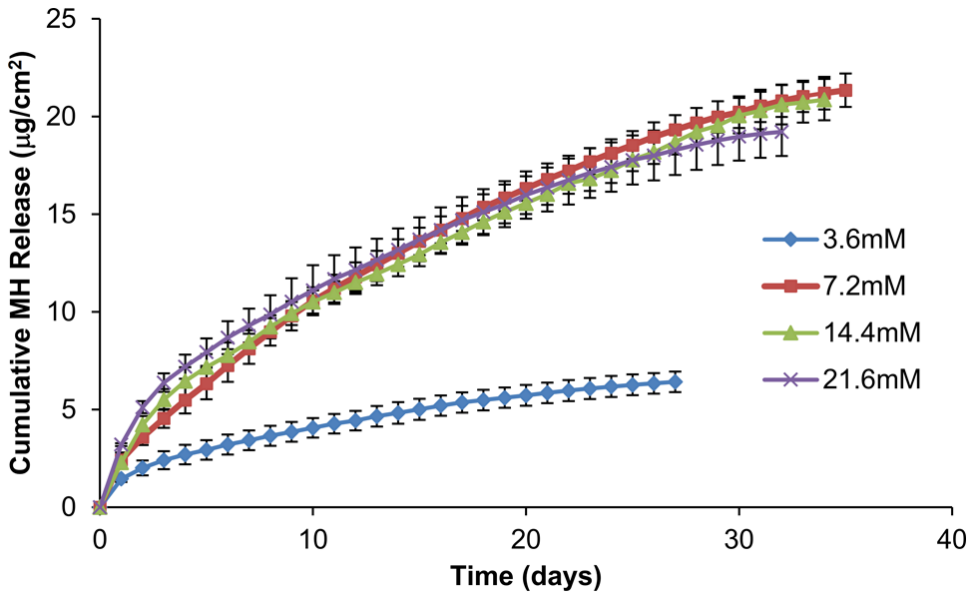
Effect of Ca^2+^ concentration on MH release from (DS+Ca^2+^/MH+Ca^2+^/GA+Ca^2+^)_8_ LbL films. Data shown are average ±STD (n = 3).

We found that the concentration of Ca^2+^ ions in the coating solutions affect MH loading and release (Figure 2). Increasing Ca^2+^ concentration from 3.6 to 7.2 mM increased drug loading in 8 trilayers of LbL films from 6.4 to 21.4 µg/cm^2^, and prolonged the duration of MH release from 27 to 35 days. However, further increasing Ca^2+^ concentration above 7.2 mM slightly reduced drug loading and the duration of MH release in a dose-dependent fashion, suggesting that 7.2 mM is the optimum concentration for high drug loading and extended release. We speculate that high concentration (above 7.2 mM) of Ca^2+^ ions in the coating solution can overcome the attraction of MH by Ca^2+^ ions in the LbL film and causing detachment of MH from the coating. Thus, 7.2 mM Ca^2+^ was used in the following studies.

### Effect of Ca^2+^ incorporation in MH and GA layers on MH release kinetics

**Figure 3:**
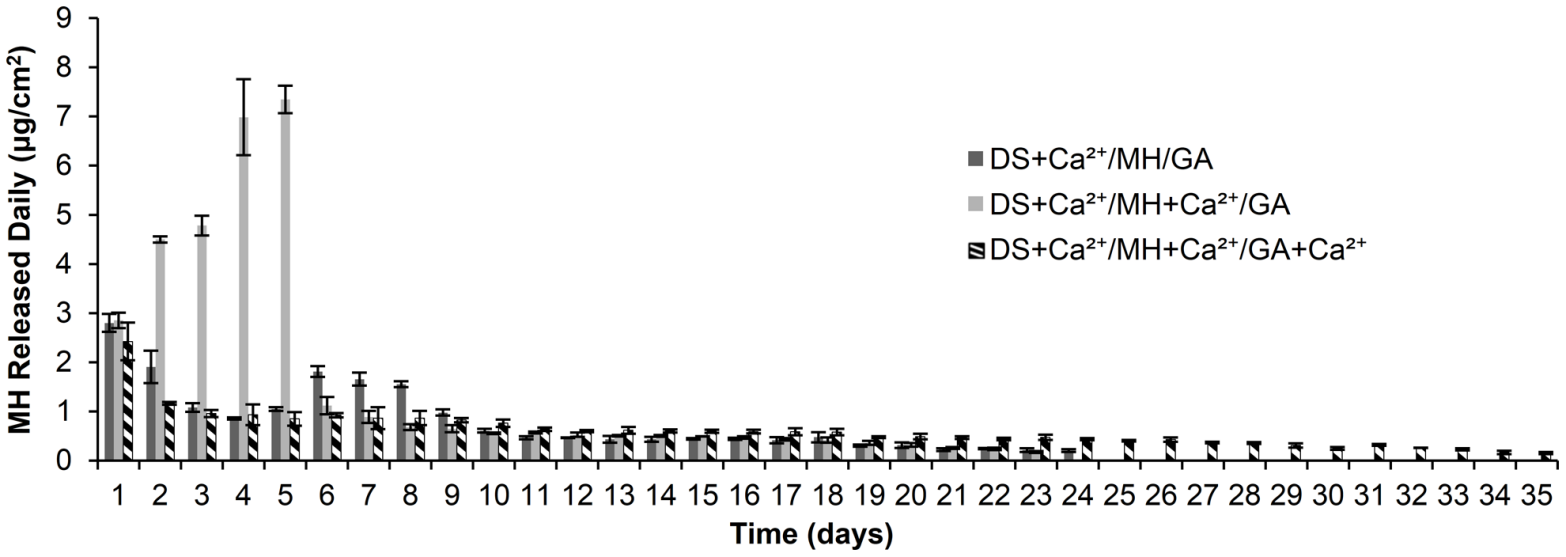
Effect of Ca^2+^ incorporation on MH release from (DS+Ca^2+^/MH+Ca^2+^/GA+Ca^2+^)_8_ LbL films. Data shown are average ±STD (n = 3).

Since MH loading and release are mediated by Ca^2+^ binding and gelatin also has Ca^2+^ binding affinity [43], incorporation of Ca^2+^ in MH and GA layers may affect the release kinetics. As shown in Figure 3, for (DS+Ca^2+^/MH/GA)_8_ multilayer films there was an initial burst release during the first two days, and a second burst release between 6–8 days, followed by a slow, stable release until 24 days. Adding Ca^2+^ in DS and MH layers, but not GA layers resulted in a high initial burst during the first 5 days, with accelerating release of MH during this period, followed by a slow, stable release up to 21 days. Both release profiles indicate a destabilization of MH binding in the LbL films during release. These release profiles may be beneficial for the treatment of acute or recurrent pathological conditions where high initial burst or pulsed release is desired. However, stable drug release is required for the treatment of chronic conditions. We found that addition of Ca^2+^ in GA layers eliminated the destabilization effect. MH release from (DS+Ca^2+^/MH+Ca^2+^/GA+Ca^2+^)_8_ films showed a low initial burst release, followed by slow, stable release for over 35 days, suggesting a more stable MH binding. Incorporation of Ca^2+^ in the GA layers may increase the amount of Ca^2+^ ions in the LbL film and thereby increase the capacity of the film to attract MH. Control over release behavior using Ca^2+^ binding may allow its application to various pathological situations.

### Effect of initial loading on MH release

**Figure 4:**
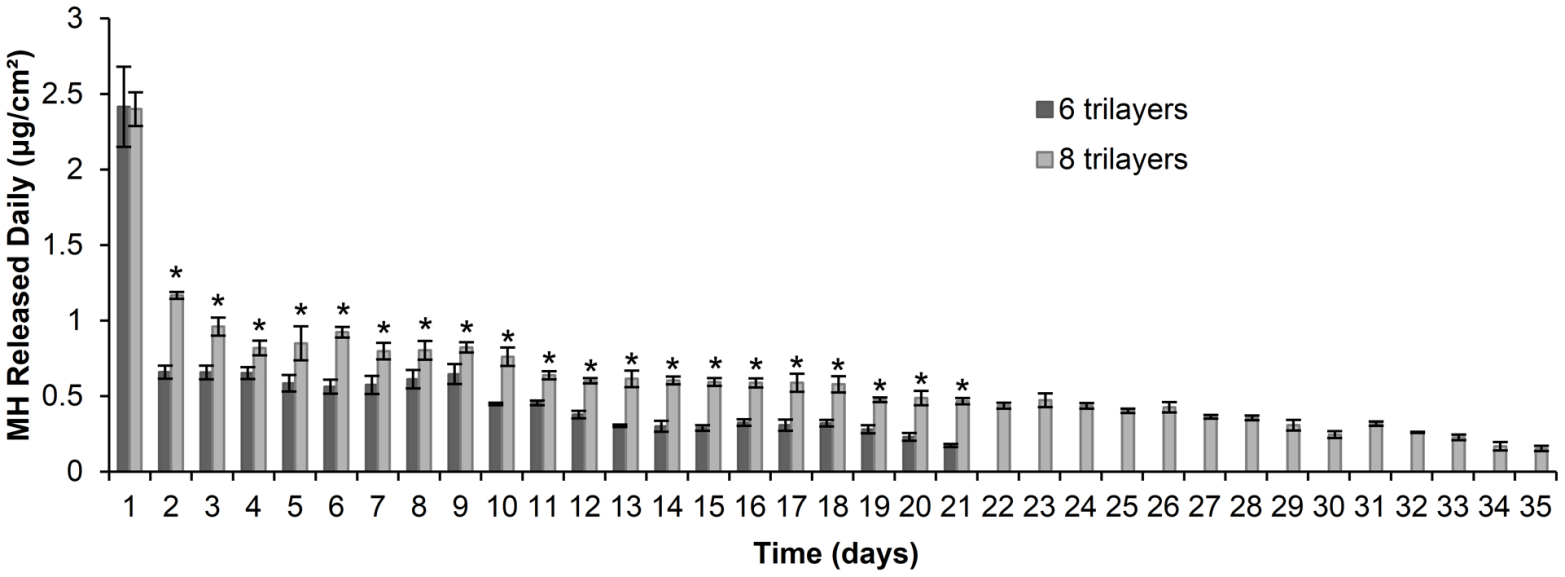
Daily MH release from 6 and 8 trilayers of (DS+Ca^2+^/MH+Ca^2+^/GA+Ca^2+^). *, *P*<0.05 compared with release from 6 trilayers. Data shown are average ±STD (n = 3).

MH release can also be tailored by adjusting its initial loading (number of trilayers). Increasing the number of trilayers of (DS+Ca^2+^/MH+Ca^2+^/GA+Ca^2+^) LbL films from 6 to 8 significantly increased the amount of MH released every day except the first day, and prolonged the release time from 21 to 35 days (Figure 4).

### Effect of pH on MH release

**Figure 5:**
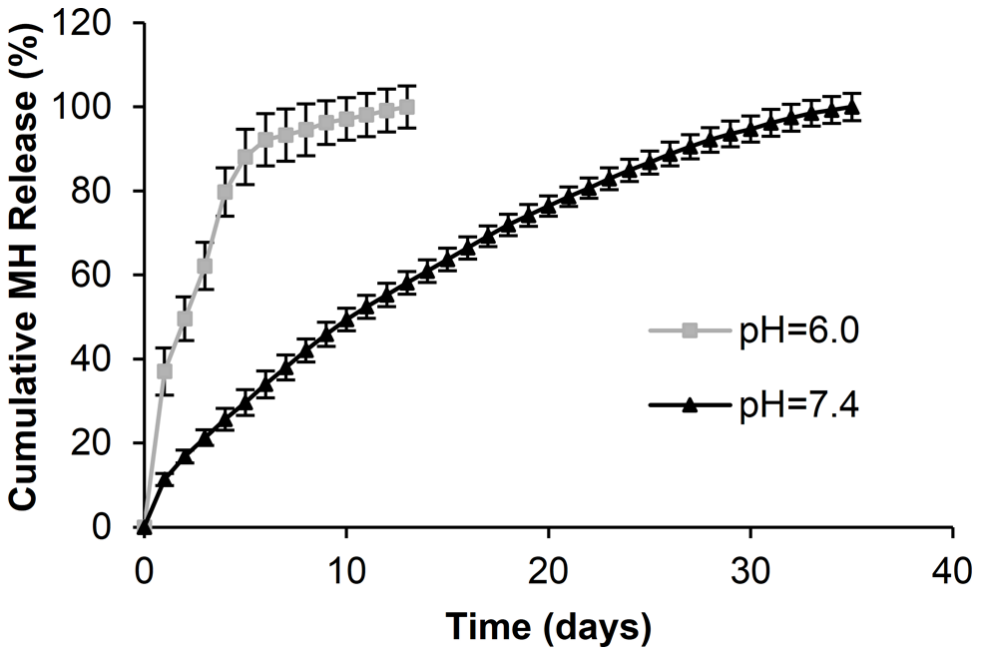
Effect of pH on MH release from (DS+Ca^2+^/MH+Ca^2+^/GA+Ca^2+^)_8_ LbL films. Data shown are average ±STD (n = 3).

To simulate pathological and physiological conditions, HBSS with pH of 6.0 and 7.4 were used as the release media. MH release from (DS+Ca^2+^/MH+Ca^2+^/GA+Ca^2+^)_8_ multilayer films only lasted 13 days at pH 6.0, with a high initial burst release, versus 35 days of sustained release and a small burst release at physiological pH (Figure 5), suggesting that Ca^2+^ binding-mediated MH release is pH-sensitive. Studies have shown that the binding affinity of tetracycline for Ca^2+^ decreases with pH [31], [32]. Thus, reduced pH can weaken the chelation between MH and DS-bound Ca^2+^ and facilitate MH release. This result suggests that in addition to detachment of MH-Ca^2+^ chelates from DS, MH release can also be mediated by dissociation of MH from the chelates. This property will potentially enable ‘smart’ drug release in response to infection and/or inflammation-induced tissue acidosis at the implant-tissue interface.

### Bioactivity of released MH

**Figure 6:**
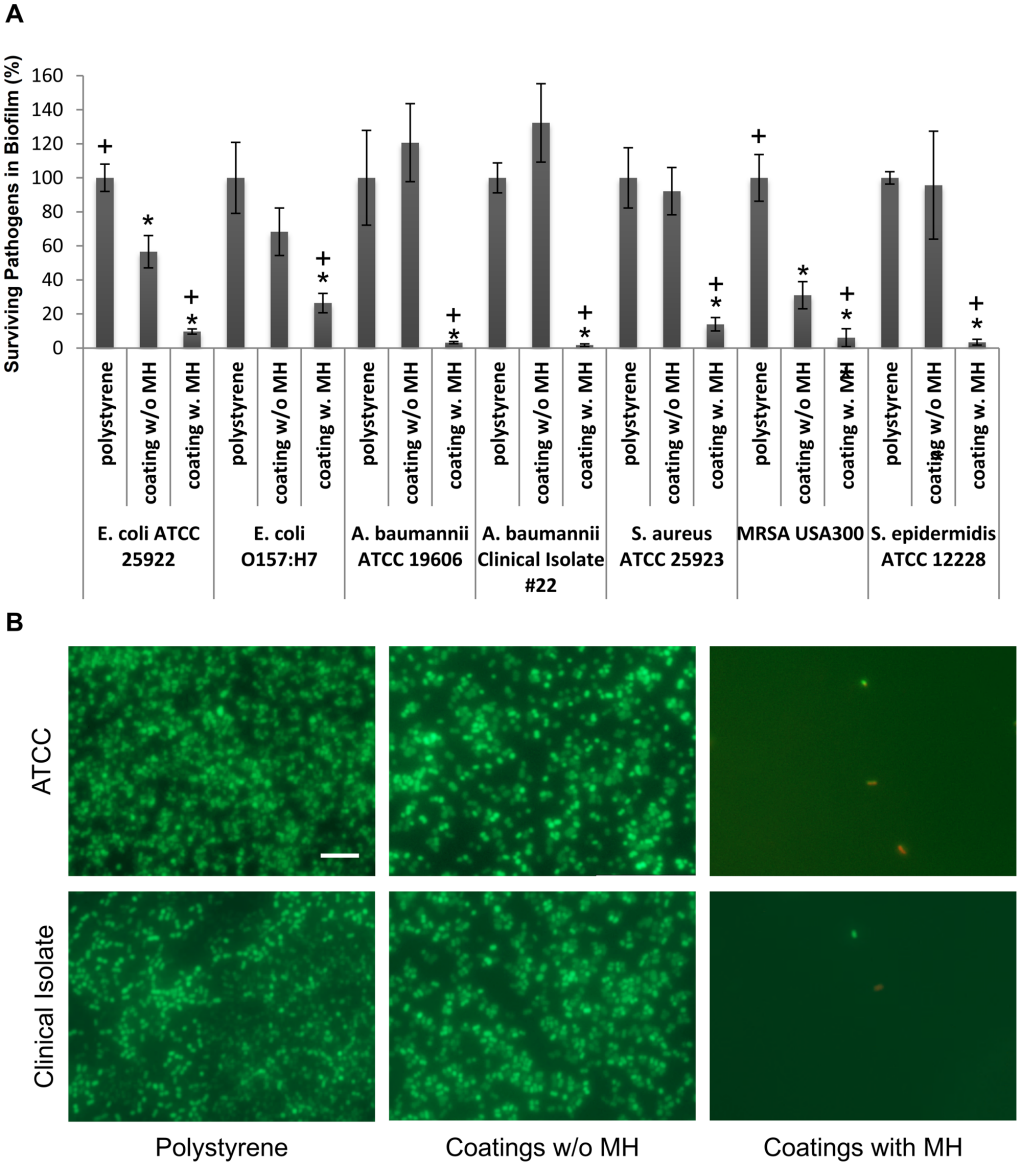
The anti-biofilm activity of (DS+Ca^2+^/MH+Ca^2+^/GA+Ca^2+^)_8_ LbL film. (A) XTT assays to detect surviving bacteria demonstrate inactivation of bacteria in biofilms as a result of MH release from coatings. Significant anti-biofilm activity was observed in the wells coated with films containing MH. *, *P*<0.05 compared with uncoated control (polystyrene); +, *P*<0.05 compared with coatings without MH. Data shown are average ±STD (n = 4). (B) Fluorescent images of *A. baumannii* from ATCC and clinical isolate cultured on uncoated polystyrene, coatings with and without MH. The cells were stained with “live” SYTO 9 stain (green) and “dead” propidium iodide stain (red). Biofilm formation was eliminated on coatings with MH. Scale bar  = 10 µm.

To evaluate the antibacterial and anti-biofilm efficacies of this novel coating, we selected seven bacterial species that are known to be involved in implant-associated infection, including a multi-drug resistant *A. baumannii* clinical isolate that was locally isolated from a hospitalized patient having invasive infection. *A. baumannii* is one of the antimicrobial-resistant bacilli that are most difficult to control and treat in clinical settings and is a strong biofilm producer [44]. Figure 6A demonstrates that coatings incorporating MH significantly inhibited biofilm formation by all seven virulent pathogens, leaving negligible pathogens in the biofilm. Although the body's natural defense system is often ineffective to remove bacterial within a biofilm, it is effective to clear adherent bacteria once the granulocytes can penetrate the biofilm. Thus, these low numbers of surviving bacteria are often cleared easily by the body's defense system since no biofilm was formed [45]. *A. baumannii* from ATCC and clinical isolate were stained with LIVE/DEAD BacLight Bacterial Viability Kit. As shown in Figure 6B, *A. baumannii* from both sources formed biofilm on uncoated polystyrene and coatings without MH, whereas only a few bacteria were present on coatings with MH.

**Figure 7:**
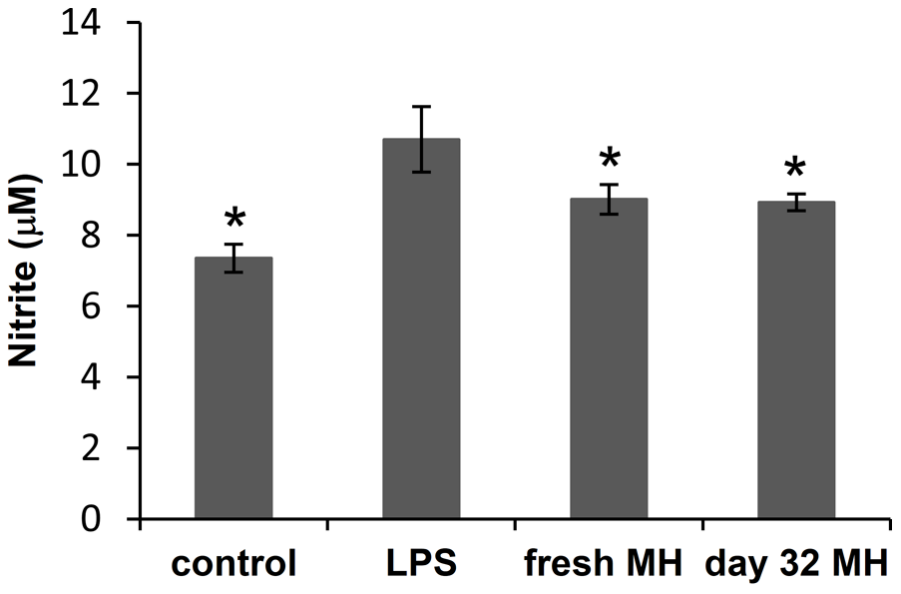
The anti-inflammatory activity of MH released from (DS+Ca^2+^/MH+Ca^2+^/GA+Ca^2+^)_8_ LbL film. NO production by macrophages treated with LPS, LPS and fresh MH (1 µg/ml), or LPS and MH released on day 32 (diluted to 1 µg/ml). NO level in cells without any treatment was used as control. *, *P*<0.05 compared with LPS-treated culture. Data shown are average ±STD (n = 3).

The anti-inflammatory potency of released MH was studied using RAW264.7 macrophages. LPS was used to stimulate the macrophages to the inflammatory phenotype, which is marked by upregulation of nitric oxide (NO), a potent inflammatory mediator and cytotoxic molecule [46]. Studies have shown that MH as an anti-inflammatory drug can inhibit the activation of macrophages and NO production [47], [48]. MH released during a 24 h period on day 32 was diluted to 1 µg/ml and added to LPS-treated macrophage cultures. Cultures treated with LPS and 1 µg/ml fresh MH were used as controls. Released MH significantly inhibited LPS-induced NO production to the same degree as freshly prepared MH (Figure 7), suggesting that released MH retained the same bioactivity as fresh MH even after one month of release.

### Cytotoxicity of the LbL coatings

The cytotoxicity of the coatings was evaluated using NIH 3T3 fibroblasts (Figure S1). Cells treated with release media collected from coatings with or without MH showed similar viability as cells without any treatment (Figure S1A). The morphology of fibroblasts incubated with release medium was comparable to that of untreated control (Figure S1B–D). The release media contain released DS and GA (data not shown). This result suggests that released MH and coating materials are not cytotoxic.

## Conclusions

In conclusion, a Ca^2+^ binding-mediated drug delivery mechanism was developed in this study. This mechanism allows for efficient loading and sustained release of MH, an effective antibiotic and anti-inflammatory drug, from nanoscale thin hydrophilic LbL films. The release kinetics of MH is mediated by calcium binding and can be controlled by varying initial loading, Ca^2+^ concentration, and adding Ca^2+^ into different layers, facilitating easy customization of versatile coatings with tailored release kinetics. This drug delivery system also allows pH-responsive MH release, enabling ‘smart’ drug release in response to the severity of infection and inflammation. This versatile drug delivery system will be especially useful for infection/inflammation-susceptible medical devices such as transcutaneous catheters, neural electrodes, and biosensors. In addition, the Ca^2+^ binding-mediated drug delivery mechanism can potentially be applied to other drugs and/or polymers with high Ca^2+^ binding affinity, enabling its use in a variety of biomedical applications.

## Supporting Information

Figure S1 **Cytotoxicity assay.** (A) Cell viability under different treatments. Data shown are average ±STD (n = 3). Phase contrast images show the morphology of 3T3 fibroblast cells (stained with cresyl violet) following 24 h treatment with (A) no treatment control, (B) release medium from LbL films containing MH, and (C) release medium from LbL films without MH. Scale bar  = 100 µm.
(TIF)
