# Supplementary figures and images for: Calcium Binding-Mediated Sustained Release of Minocycline from Hydrophilic Multilayer Coatings Targeting Infection and Inflammation
Source: PLoS One. 2014 Jan 7;9(1):e84360. doi: 10.1371/journal.pone.0084360 (PMC3883660; doi:10.1371/journal.pone.0084360)

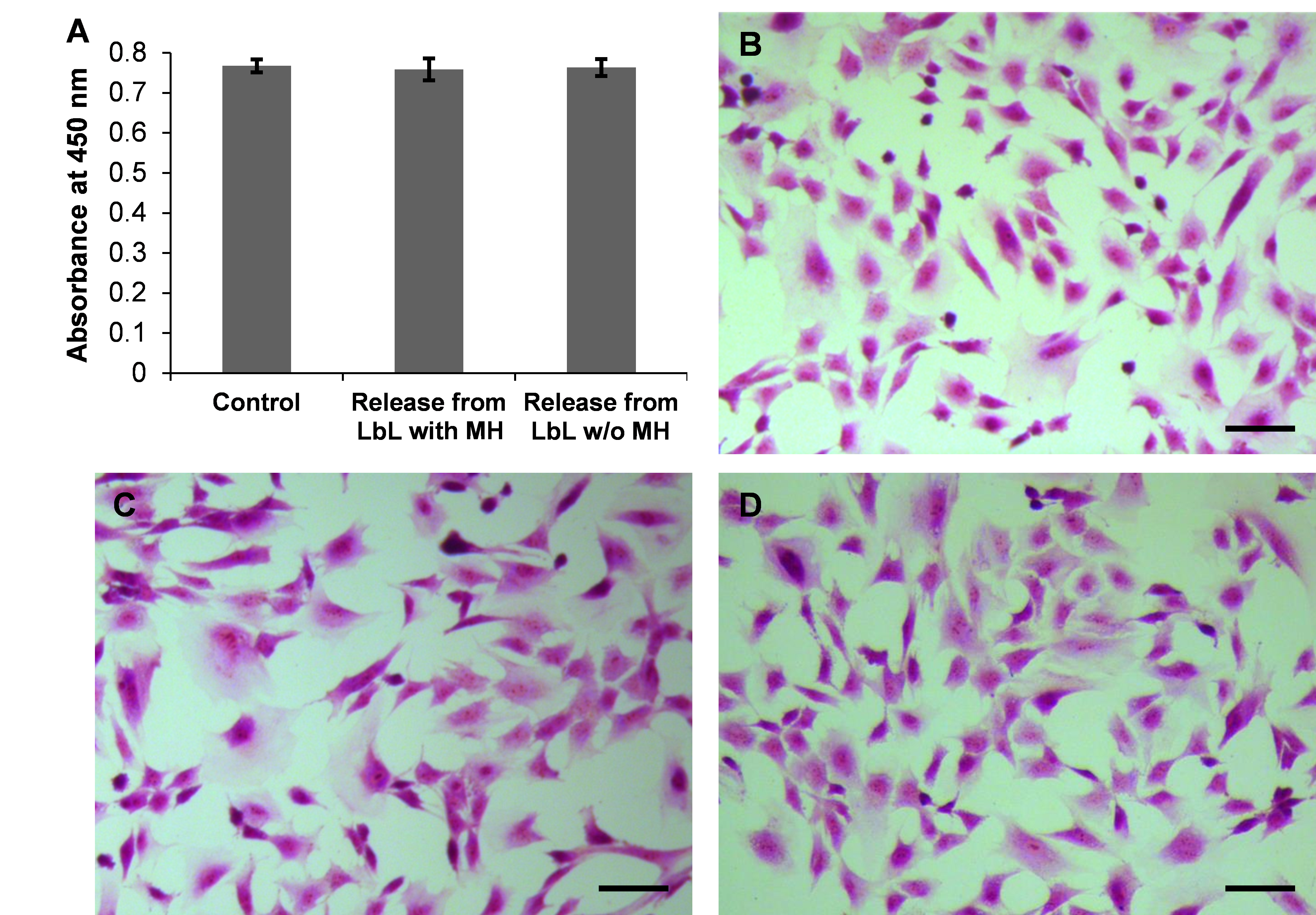

Supplement: Figure S1 — Cytotoxicity assay. (A) Cell viability under different treatments. Data shown are average ±STD (n = 3). Phase contrast images show the morphology of 3T3 fibroblast cells (stained with cresyl violet) following 24 h treatment with (A) no treatment control, (B) release medium from LbL films containing MH, and (C) release medium from LbL films without MH. Scale bar = 100 µm. (TIF) [file pone.0084360.s001.tif]
